# Rising Obesity-Related Hospital Admissions among Children and Young People in England: National Time Trends Study

**DOI:** 10.1371/journal.pone.0065764

**Published:** 2013-06-12

**Authors:** Jessica D. Jones Nielsen, Anthony A. Laverty, Christopher Millett, Arch G. Mainous III, Azeem Majeed, Sonia Saxena

**Affiliations:** 1 Department of Primary Care and Public Health, School of Public Health, Imperial College London, London, United Kingdom; 2 Department of Family Medicine, Medical University of South Carolina, Charleston, South Carolina, United States of America; Old Dominion University, United States of America

## Abstract

**Objective:**

To describe the trends in hospital admissions associated with obesity as a primary diagnosis and comorbidity, and bariatric surgery procedures among children and young people in England.

**Design:**

National time trends study of hospital admissions data between 2000 and 2009.

**Participants:**

Children and young people aged 5 to 19 years who were admitted to hospital with any diagnosis of obesity.

**Main outcome measures:**

Age- and sex-specific admission rates per million children.

**Results:**

Between 2000 and 2009, age- and sex-specific hospital admission rates in 5–19 year olds for total obesity-related diagnoses increased more than four-fold from 93.0 (95% CI 86.0 to 100.0) per million children to 414.0 (95% CI 410.7 to 417.5) per million children, largely due to rising admissions where obesity was mentioned as a co-morbidity. The median age of admission to hospital over the study period was 14.0 years; 5,566 (26.7%) admissions were for obesity and 15,319 (73.3%) mentioned obesity as a comorbidity. Admissions were more common in girls than boys (56.2% v 43.8%). The most common reasons for admission where obesity was a comorbid condition were sleep apnoea, asthma, and complications of pregnancy. The number of bariatric surgery procedures has risen from 1 per year in 2000 to 31 in 2009, with the majority were performed in obese girls (75.6%) aged 13–19 years.

**Conclusions:**

Hospital admission rates for obesity and related comorbid conditions have increased more than four-fold over the past decade amongst children and young people. Although some of the increase is likely to be due to improved case ascertainment, conditions associated with obesity in children and young people are imposing greater challenges for health care providers in English hospitals. Most inpatient care is directed at dealing with associated conditions rather than primary assessment and management of obesity itself.

## Introduction

Childhood obesity has become a global epidemic and continues to be a worldwide health concern in many developing and developed countries including the U.K. and the U.S. [1], [2], [3], [4] Although the steep rise in childhood obesity in England appears to be levelling off, national surveys in England suggest that around three in ten boys and girls (31% and 29% respectively) aged 2 to 15 years are overweight [5] and 14 to 20% of children aged 2 to 15 years are obese. [6] Good evidence from birth cohorts shows obesity is associated with serious illness and health-related problems during childhood. [7], [8], [9] Children who are obese are at increased risk of developing serious co-morbid conditions including Type II diabetes, asthma and sleep apnoea [10], [11] and follow trajectories into adulthood where the health consequences associated with obesity are more severe than in people who develop obesity later in life. [12], [13] More recently, emerging evidence suggests many severely obese children already have cardiovascular risk factors and clinicians and other hospital staff have come to recognize that obesity is an important risk factor among this patient population [14].

Several prior studies have examined the health consequences and the costs associated with obesity among adults in both the U.K. and the U.S. [15], [16], [17] The number of adult patients admitted to National Health Service (NHS) hospitals with a primary diagnosis of obesity has increased 10-fold over the last decade. [5] As obesity in childhood becomes more common, the health consequences may be witnessed earlier in the life course, which could translate into large increases in demand for health care services.

The spiralling costs of treating obesity-related conditions in U.S. adolescents have been well documented. [18], [19], [20], [21], [22] In the U.K., where access to healthcare is freely accessible, the impact of managing and treating the growing epidemic of obesity in young people on the NHS has not yet been estimated. The aim of this paper was to describe the trends in hospital admissions that are associated with obesity as primary diagnosis and comorbidity, and bariatric surgery procedures among children and young people aged 5 to 19 years in English hospitals between 2000 and 2009.

## Methods

### Data Sources

The Hospital Episodes Statistics (HES) database [23] records all finished consultant episodes of all admissions in the National Health Service (NHS) hospital care in England, including treatment funded by the NHS but performed in private hospitals. Episodes are defined as the time period during which an admitted patient is under the care of one NHS hospital consultant during a single hospital admission. The main reason for admission or ‘primary diagnosis’ is coded using the International Classification of Diseases version 10 (ICD-10) codes, along with up to 19 subsequent codes or ‘secondary diagnoses’ to denote comorbid conditions. In addition, the HES database includes information on patient characteristics and clinical procedures in addition to geographic and administrative data. [24] We had approval to use the HES data from the National Health Service Information Centre, and formal ethical approval is not needed for to use this routinely collected, anonymised data.

### Admission for Treatment of Obesity and Obesity-related Conditions

For the purposes of this study, we used HES data to examine elective or emergency obesity admissions in all children and young people aged 5 to 19 years between April 2000 and March 2010. Obesity admissions were identified using ICD-10 codes *E660 (obesity due to excess calories), E661 (drug induced obesity), E662 (morbid or severe obesity), E668 (other obesity),* and *E669 (obesity, unspecified)*. We defined total obesity admissions as comprising both primary and secondary diagnoses of obesity. Admissions for obesity were those where the primary diagnosis was for management of obesity. We defined obesity-related admissions as those where the main reason for admission was for any condition other than obesity and obesity was also listed in a secondary diagnostic field as a comorbidity. We created three age bands: 5–9, 10–14, and 15–19 years, reflecting key stages of childhood development and to allow comparison with other studies. Children aged less than 5 years were excluded since we considered inclusion of admissions to hospital in this age group might introduce bias towards those admitted for investigation or treatment of rare congenital abnormalities (*n* = 1,891).

Using a combination of primary diagnostic codes specific to obesity and surgical procedures codes, we also identified children and young people who underwent bariatric surgical procedures for the management of obesity during the study period. The procedures examined in this study were used from those reported in the National Obesity Observatory paper on bariatric surgery for obesity [25].

We used the Agency for Healthcare Research and Quality’s Clinical Classification System (CCS) codes [26] to extract the most common primary diagnoses when obesity is a secondary diagnosis. The CCS aims to group ICD-10 codes into more refined classifications by collapsing ICD-10 codes into 259 clinically meaningful categories, which have been extensively used in other studies [22], [27].

We obtained mid-year population estimates for England for 2000 to 2009, stratified by 3 age groups and sex from the Office for National Statistics (ONS) [28] to compare time trends in admission rates. We calculated age- and sex-specific admission rates per million children and associated 95% confidence intervals (CIs) for all years and stratified by age by using ONS population data as a denominator. We tested differences in admission rates using the chi-square test and agreed a statistical significance level of *p*≤0.05 for all hypothesis testing. All data analysis was conducted using SPSS software version 19.0 (SPSS, Inc., Chicago, IL, USA).

## Results

### Hospital Admissions

We found the majority of admissions over the entire study period of 2000 to 2009 were for obesity as a comorbidity (15,319, 73.3%), compared to 5,566 (26.7%) admissions which were for obesity itself (Table 1). The median age of children and young people admitted to hospital for obesity was 13.0 years, and 14.0 years among those with obesity as comorbidity. Overall admissions were more common in girls than boys (56.2% v 43.8%) and increased with age from 18.7%, 40.1% and 41.2% for age groups 5–9, 10–14 and 15–19 years respectively.

**Table 1:** Demographic characteristics of children and young people admitted with obesity-related diagnosis over the entire study period, by primary or secondary diagnosis, 2000 to 2009.

	Admissions for obesity, n (%)	Admissions with obesity as a comorbidity, n (%)	Total obesity-related admissions, n (%)
Age (median)	13.0 years	14.0 years	14.0 years
Sex			
Boys	2,444 (43.9)	6,710 (43.8)	9154 (43.8)
Girls	3,120 (56.1)	8,611 (56.2)	11731 (56.2)
Age group			
5–9 years	1,249 (22.4)	2,664 (17.4)	3913 (18.7)
10–14 years	2,915 (52.4)	5,446 (35.6)	8361 (40.1)
15–19 years	1,402 (25.2)	7,209 (47.0)	8611 (41.2)
Ethnic group			
White	3,317 (59.6)	10,197 (66.6)	13514 (64.7)
South Asian	590 (10.6)	1,121 (7.3)	1711 (8.2)
Black	246 (4.4)	512 (3.3)	758 (3.6)
Mixed	141 (2.5)	263 (1.7)	404 (1.9)
Other ethnicity	115 (2.1)	266 (1.7)	381 (1.8)
Not known	1,157 (20.8)	2,960 (19.3)	4117 (19.8)
Total	5,566	15,319	20,885

**Figure 1:**
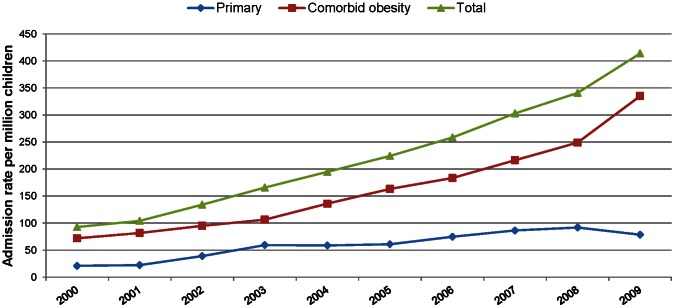
Trends in age and sex-specific hospital admission rates per million children among children and young people ages 5 to 19 years, for obesity and where obesity was a comorbidity, 2000–2009.

Hospital admission rates for obesity increased almost four-fold from 21.0 (95% CI 17.5 to 24.5) per million children in 2000 to 78.8 (95% CI 77.0 to 80.6) in 2009. Admission rates for obesity as a comorbidity increased from 70 (95% CI 65.6 to 78.4) per million children in 2000 to 335.3 (95% CI 332.3 to 338.3) per million children in 2009 (Figure 1). The increase for obesity as a comorbidity has continued year on year, but in the last year, the rate for admissions due to obesity declined by 14.4% from 92.0 (95% CI 90.3 to 93.7) per million children in 2008 to 78.8 (95% CI 77.0 to 80.6) in 2009. This decline was significant statistically (*p*<0.001). Table 2 shows the age- and sex-specific rates of hospital admission for obesity as a primary and secondary diagnosis from 2000 to 2009. From Table 2 we can see that the overall admission rates have risen in all age groups for both boys and girls. In each year between 2000 and 2009, the numbers and rates of hospital admissions for a diagnosis of obesity were higher for girls than for boys.

**Table 2:** Age- and sex-specific hospital admission rates per million children for obesity as a primary diagnosis and where obesity was a comorbidity, 2000–2009.

Age (Years)	Obesity Diagnosis	Year									
		2000	2001	2002	2003	2004	2005	2006	2007	2008	2009
Boys overall number admissions		421	496	621	660	863	1016	1095	1214	1337	1424
5–9	Primary	16.6	11.9	22.1	33.9	39.5	42.7	49.6	46.4	55	51.2
	Secondary	49.1	53.2	62.6	60.8	133.4	160.4	124.7	135.2	127.1	148.2
10–14	Primary	29.9	30.2	68.2	52.8	82.7	94.1	108.4	114.9	131.9	84.9
	Secondary	84.3	121.2	117.8	136.8	141.3	172.2	253.5	242.5	283.6	321.3
15–19	Primary	13.7	16	19.3	23.6	32.7	26	37.2	65.9	56.1	66.4
	Secondary	69.2	74.4	90.2	94.9	101.2	134.2	110.5	155	191	230.5
Boys OverallMid-year Population Estimates (in 1,000s)		4,797.0	4,815.8	4,860.5	4,885.7	4,881.2	4,849.3	4,817.4	4,776.9	4,729.8	4,708.7
Girls overall number admissions		451	485	651	918	989	1107	1330	1608	1810	2382
5–9	Primary	23.9	27.6	32.6	54.4	51.6	48.2	55.3	57.7	66.1	60.0
	Secondary	40.1	44.7	55.9	63.1	72.6	78.5	84.1	80.5	97	122.2
10–14	Primary	29.5	33.6	54	149.8	100.8	113.1	127.4	160	161.6	135.1
	Secondary	67.9	88.6	106.8	103.6	145.4	170.7	202.9	206.4	214.6	286.5
15–19	Primary	11.6	14.8	37.8	43.9	44.8	43.6	73.1	73.8	82.8	75.7
	Secondary	124.4	107.6	135.8	176.7	220.4	257.6	315.8	456.5	548.7	859.1
Girls OverallMid-year Population Estimates (in 1,000s)		4,580.0	4,588.0	4,603.5	4,622.9	4,625.5	4,607.0	4,565.2	4,535.6	4,501.5	4,482.7

### Bariatric Surgery Procedures

**Figure 2:**
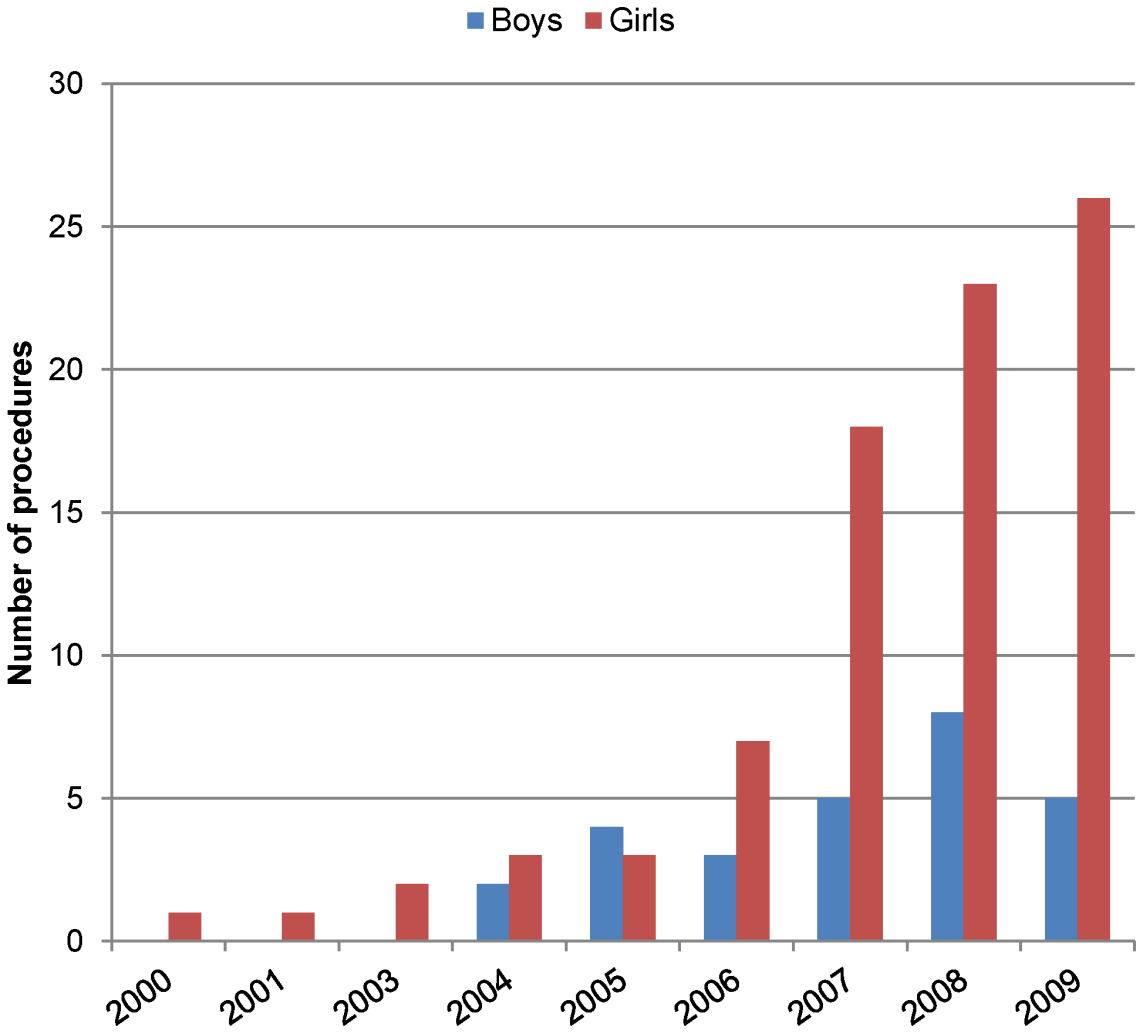
Number of hospital admissions for bariatric surgery for children and young people ages 13 to 19 years by sex, 2000 to 2009.

Of the 20,885 children and young people admitted to hospitals in England during the study period, 111 patients had a bariatric surgery procedure performed during 2000 and 2009 and the number increased from one procedure in 2000 to 31 in 2009. Of those 111 patients, 109 were in the 15–19 years age group and only two were in the 10–14 years age group. The majority of these procedures (75.6%, *n* = 84) were performed in girls and the median age was 18 years (Figure 2). Partitioning of stomach using a band was the most commonly performed bariatric surgical procedure, accounting for 36% (*n* = 34) of procedures during the study period (data not shown).

### Hospital Admission Rates by Clinical Classification System (CCS) Diagnoses

**Figure 3:**
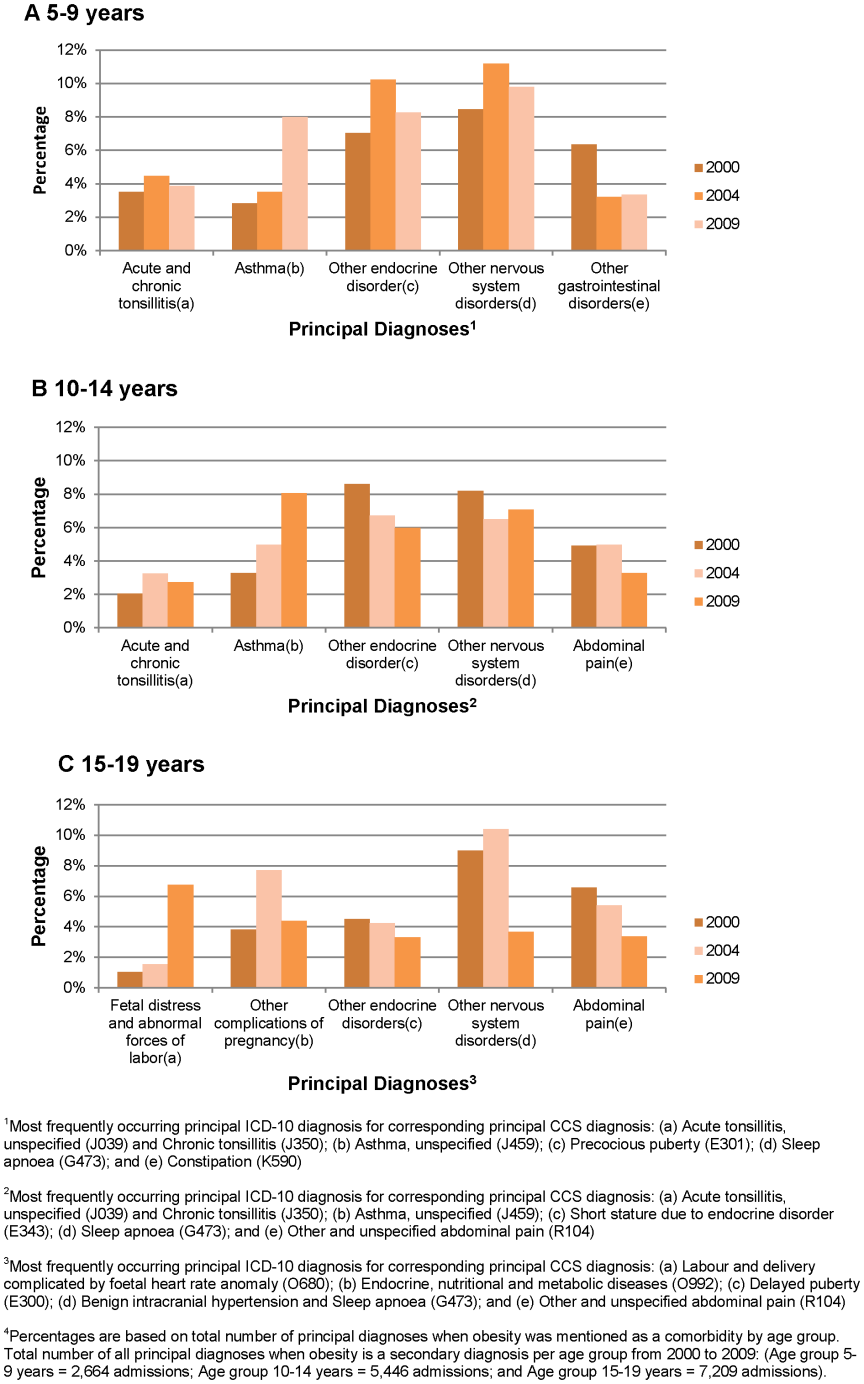
Percentage of obese children admitted to hospital by most common reasons for admission, 2000, 2004 and 2009.

The most common primary diagnoses (CCS codes) where obesity was a comorbidity were aggregated into five categories for each age group (see Figure 3). The proportion of the 5 most common CCS diagnoses varied over time and by age. Across all age groups and years, the most common CCS category diagnosis was ‘other nervous system disorders’, which was mainly caused by sleep apnoea (ICD-10 code *G473*) as a primary reason for admission, while the second most common was asthma (see Figure 3).

Figure 3 shows for three years that among admissions for patients in age groups 5–9 years, the most common CCS diagnosis in was ‘other endocrine disorders’ as one of the most common reasons for hospital admissions where obesity was a secondary diagnosis. For patients in the 15–19 years age group, the CCS diagnoses ‘foetal distress and abnormal forces of labour’ and ‘other endocrine disorders’ as the primary reason for admission increased markedly over the study period. Other conditions show a mixed pattern over time.

## Discussion

We identified an almost four-fold increase in admissions for obesity, and a more than five-fold increase in hospital admissions where obesity was comorbidity amongst children and young people in England from 2000 to 2009. The largest proportionate increase was among young people aged 15 and 19 years. Three quarters of obese children and young people (72.8%, *n* = 16,571) were admitted with obesity as a comorbidity rather than as the main reason for admission. Rates for admissions with obesity as comorbidity were consistently higher in girls than in boys over the period of the study. The most common reasons for admission where obesity was comorbidity were sleep apnoea, asthma, and complications of pregnancy. The number of bariatric surgery procedures has risen from one per year in 2000 to 31 in 2009, with the majority performed in girls (75.6%) aged 13–19 years.

### Strengths and Weaknesses of the Study

This is, to our knowledge, the first study to report national time trends in hospital admissions with an obesity-related diagnosis in children and young people living in England. The strength of using HES data are its large nationally representative population-based sample size and standardized data collection. Although our estimates of diseases associated with obesity are likely to be conservative in absolute terms, the data from this 9-year period reflect health care providers’ unique position on having a major impact on the obesity epidemic through improved case ascertainment and treatment of obesity among children and young people living in the U.K. Over the period of this study, case ascertainment is likely to have improved due to the heightened awareness of obesity in general, as well as responses to newly implemented healthcare initiatives over the last decade and newly published guidelines for England in 2006. [29] Hence, our study is not aimed at identifying the true prevalence of obesity in children admitted to hospital but to describe the profile of obesity associated admissions. Nevertheless, the study does have several important limitations.

As with all large clinical databases, the quality of HES data is reliant on the accuracy and consistency of coding. [30] Clinicians are asked to record only significant diagnoses summarising the main reason for admission as primary diagnosis and also to record comorbid conditions that may contribute to their condition or treatment episode as secondary diagnoses. Measurements such as body mass index, weight and height are not recorded in HES data and so we were unable to examine trends in BMI among children admitted to hospital. This may lead to selective recording of obesity as a diagnosis in cases where obesity is more apparent or relevant to their episode of care. For example, obesity if present may be omitted from the discharge summary in some acute admissions where other conditions predominately leading to under ascertainment.

Our findings of a four-fold increase in obesity related conditions to hospital are unlikely to be explained by rising trends in obesity incidence alone. National surveys and measurement programmes in children suggest the prevalence of childhood obesity continues to increase each year, although recent studies indicate a levelling off in recent years. [31], [32], [33] Although the numbers of admissions are small compared to the admission rates of other common childhood diseases, the increases are substantial with more than a four-fold increase over the ten-year study period which is similar to findings of other developed countries. A U.S. study [22] reported an increase of 23.9% and 11.5% per year in hospitalisations when obesity was a primary and secondary diagnosis for children and young people aged 2–19 years from 1999 to 2005. More recent studies have documented a doubling in childhood obesity-associated admissions rates between 2000 and 2009, [34] which is a more conservative estimate than the near quadrupling reported here.

Additionally, we found that patients undergoing bariatric procedures were mainly girls and the number of these procedures increased substantially during our study period. The increase in bariatric surgery among adults in England has been described as exponential, [35] and this similar increase among young people suggests that there may be a growing consensus of the need to intervene early in cases where young people are obese. While the number of these surgeries started from a very low base of one in the year 2000, and the numbers are still lower than U.S. estimates. [22] The increase is large and suggests that England may be moving towards the greater number of these procedures seen in the U.S.

We already know that obesity is associated with a variety of health problems in later life. However, the results of this study indicate that these health consequences may be presenting earlier in the life course and are being recognized sooner by English health care providers. This represents serious ill-health for the patients involved, but also a large challenge for health systems and for the health care providers that work within them. Children who are overweight as young as four years old are likely to continue to be overweight or obese as they get older, [36] which suggests that health care providers continue to recognise obesity earlier and may be advised to counsel even young overweight children to adopt healthier lifestyles. The main diseases where obesity was mentioned as a comorbidity were asthma and sleep apnoea. Both of these diseases have been linked to obesity among children and young people in other studies [37], [38] and the severity and rate at which they occur have been mainly linked to adult obesity and higher rates of mortality in the future. [7], [8], [39], [40].

### Implications and Unanswered Questions

Our study highlights that obesity admission rates may be attributable to more case finding and active assessment and treatment as a result of greater awareness among health professionals or better coding of health service activity.” [41] It is highly likely that our findings are the net result of combination of these effects. Obesity-related conditions are expensive to treat but early diagnosis in primary care settings can alert families to work with clinicians to address and treat conditions before complications set in. Bariatric surgery remains an effective intervention but U.S. reports suggest it is underutilized by the majority of children who meet the eligibility criteria for this procedure. [42] We recommend that future research should examine any mismatch between the uptake of surgery and eligibility.

Our findings of increasing admissions where obesity is a comorbidity among pregnant teenage girls may be related to more active case finding in the course of assessment during pregnancy or gestational obesity and warrants further investigation. This may be an area which would benefit from better data collection in the future. The U.K. has data from the National Child Measurement Programme, which was established in 2006 to increase public and professional understanding of weight related issues and it seems likely that ascertainment is likely to improve throughout the lifetime of the programme. This improved data at a national level, along with improved coding and understanding of the importance of early diagnosis among clinical staff has the potential to improve the health of both these children and the population at large.

Better ascertainment is likely to increase associated costs of treating obesity and its sequelae, at least in the short term. Our research also highlights the need for a full economic analysis of the cost of managing and treating obesity in children and young people. This will ultimately need to be ‘weighed’ against the effectiveness of whole population public health and primary care interventions. Future research should aim to identify target groups of children with higher prevalence of obesity and investigate how this compares with those treated for obesity-related conditions in hospitals in England. Further work is also needed to investigate whether the publication of national guidelines have impacted on hospital admission patterns for obesity.

### Conclusions

Hospital admission rates for obesity have increased more than four-fold over the past decade in children and young people, particularly amongst girls and in older age groups. NHS hospitals are using more and more resources in treating conditions associated with obesity and the majority of resources are directed at dealing with admissions where obesity is a comorbidity (such as asthma and sleep apnoea) rather than primary assessment and management of obesity itself. This study highlights the need for public health action to quantify the scale of the problem more clearly through early detection and reverse recent trends in order to reduce the number of admissions caused by obesity among children and young people in England. Our findings support emerging evidence that the childhood obesity epidemic may lead to substantial problems of obesity-related disease much sooner in children and young people’s lives than previously expected. [14] With levels of admissions for obesity-related diagnoses rising, there is likely to be increasing demand on health services and also greater use of more radical interventions, such as pharmacological or surgical treatment, as part of efforts to address the increasing trend of obesity that threatens the lives of many children and young people in England and globally.
